# Metformin attenuates diabetic osteoporosis by suppressing ferroptosis via the AMPK/Nrf2 pathway

**DOI:** 10.3389/fphar.2025.1527316

**Published:** 2025-03-26

**Authors:** Yanwei Liu, Zhaoyu Fu, Xinyu Wang, Qifan Yang, Shun Liu, Dong Zhu

**Affiliations:** ^1^ Department of Orthopaedics, The First Hospital of Jilin University, Changchun, China; ^2^ Department of Emergency Surgery, The First Affiliated Hospital of Zhengzhou University, Henan Medical Key Laboratory of Emergency and Trauma Research, Zhengzhou, Henan, China

**Keywords:** metformin, diabetic osteoporosis, ferroptosis, osteoblast, Nrf2/AMPK

## Abstract

**Background:**

Ferroptosis is a critical factor in the impairment of osteoblast function in osteoporosis. Metformin (Met), a biguanide antidiabetic drug, has demonstrated anti-osteoporotic effects and has been confirmed to exert therapeutic benefits in diabetic osteoporosis (DOP). Nevertheless, the underlying mechanisms through which Met affects bone metabolism remain ambiguous.

**Objective:**

This study seeks to elucidate the function of Met in DOP and to explore the potential mechanisms through which it mediates treatment effects.

**Methods:**

*In vitro*, we utilized osteoblasts to explore the impact of Met on osteoblast differentiation and anti-ferroptosis in a high glucose and palmitic acid (HGHF) environment. *In vivo*, we developed a DOP model utilizing a high-fat diet along with streptozocin injections and evaluated the bone-protective effects of Met through micro-CT and histomorphological analyses.

**Results:**

Met inhibits HGHF-induced ferroptosis in osteoblasts, as indicated by the elevation of ferroptosis-protective proteins (GPX4, FTH1, and SLAC7A11), along with decreased lipid peroxidation and ferrous ion levels. Furthermore, Met augmented the levels of osteogenic markers (RUNX2 and COL1A1) and enhanced alkaline phosphatase activity in osteoblasts under HGHF conditions. Mechanistic investigations revealed that Met activates the AMPK/Nrf2 pathway, effectively preventing ferroptosis progression. Additionally, *in vivo* results demonstrated Met alleviates bone loss and microstructural deterioration in DOP rats.

**Conclusion:**

Met can activate the AMPK/Nrf2 pathway to prevent ferroptosis, thereby protecting against DOP.

## 1 Introduction

According to the latest data from the International Diabetes Federation, the number of adults living with diabetes worldwide amounted to 536.6 million in 2021 and is expected to increase to 783.2 million by 2045, with type 2 diabetes mellitus (T2DM) accounting for 95% of all cases (American Diabetes Association Professional Practice Committee, 2022; Sun et al., 2022). Diabetic osteoporosis (DOP), a chronic musculoskeletal complication of diabetes, primarily leads to trabecular structural deterioration and increased bone fragility (Napoli et al., 2017). Osteoporosis-related fractures exceed 9 million worldwide Each year, with the majority closely associated with DOP, posing significant challenges to human health and socioeconomic stability (Johnell and Kanis, 2006). Emerging evidence suggests that ferroptosis in osteoporosis-targeted cells, such as osteoblasts and osteocytes, represents a potential underlying mechanism of osteoporosis (Gao et al., 2022; Liu et al., 2022).

Ferroptosis, a novel mode of programmed cell death, is associated with a range of diseases, including retinal degeneration and Alzheimer’s disease (Stockwell, 2022). Its core mechanism arises from unregulated lipid peroxidation processes, which are closely dependent on the involvement of iron (Stockwell et al., 2017). Compared to other types of cell death, ferroptosis exhibits distinct biological characteristics, including iron overload, elevated lipid peroxides, and dysregulation of ferroptosis-related proteins (Stockwell, 2022). Key ferroptosis-protective proteins such as glutathione peroxidase 4 (GPx4), ferritin heavy chain 1 (FTH1), and solute carrier family 7, member 11 (SLC7A11), are regulated by the transcription factor nuclear factor erythroid 2-related factor 2 (Nrf2) (Dodson et al., 2019b; Dong et al., 2021). Under stress conditions, Nrf2 moves into the nucleus and stimulates genes featuring antioxidant response elements (Wang Y. et al., 2022). Research indicates ferroptosis contributes to the impairment of osteogenic differentiation and mineralization capacity of osteoblasts in the diabetic microenvironment (Ma et al., 2020). However, the mechanisms that ferroptosis operates in DOP have yet to be completely clarified. Therefore, targeting the ferroptosis pathway to suppression lipid peroxidation and ferroptosis may represent a promising intervention approach for osteoporosis.

Metformin (Met) is a conventional antihyperglycemic agent that is not only effective in managing T2DM but also demonstrates potential efficacy in metabolic disorder-related conditions, such as obesity (Zheng et al., 2015; Lv and Guo, 2020). One of the mechanisms of Met is the inhibition of oxidative phosphorylation, which leads to a reduction in ATP production (Pollak, 2017), subsequently activating AMP-activated protein kinase (AMPK) (Xian et al., 2021). AMPK serves as a crucial sensor of cellular energy status, influencing cellular fate by regulating the phosphorylation of various downstream substrates, such as Nrf2 (Bootman et al., 2018; Lu et al., 2021). Furthermore, studies suggest energy stress-mediated AMPK activation may be one of the mechanisms regulating ferroptosis (Lee et al., 2020). However, there is limited research on how Met reverses osteoblastic ferroptosis through AMPK signaling. Alternatively, while Met has been shown to improve osteogenesis, the relationship between this improvement and ferroptosis remains largely unexplored.

In this research, we developed a model of osteoblastic injury caused by high glucose and high palmitic acid (HGHF) *in vitro*, as well as a type 2 DOP rat model, to systematically investigate the mechanisms by which Met inhibited osteoblastic ferroptosis and improved osteoporosis in the diabetic microenvironment.

## 2 Materials and methods

### 2.1 Antibodies and reagents

Palmitic acid (PA) and control group BSA were sourced from Kunchuang Biotechnology (Xian, China). Primary antibodies for COL1A1, RUNX2, AMPK, p-AMPK, GPX4, FTH, β-ACTIN, and Histone H3 were acquired from Cell Signaling Technology (United States), while primary antibodies for SLC7A11 and Nrf2 were supplied by Proteintech Group (Wuhan, China). Streptozocin (STZ) and Met were sourced from Beyotime Biotechnology (Shanghai, China). Ferrostatin-1, AICAR, and Compound C were all obtained from MedChemExpress (Shanghai, China). Fetal bovine serum (FBS) was obtained from Cell-Box (Hong Kong, China), and the dual antibiotics (penicillin-streptomycin) were offered by NCM Biotech (Suzhou, China). Multicolor prestained protein ladders were provided by NCM Biotech (Suzhou, China) and Epizyme Biomedical Technology (Shanghai, China).

### 2.2 Animal experiments

Six-week-old SD rats were supplied by Beijing Vital River Laboratory Animal Technology Company (SCXK2021-0006), weighing 180 ± 20 g. The rats were casually allocated into two groups: control group (Con; n = 6) and T2DM model group (T2DM; n = 6). The T2DM rat model was established using an 8-week high-fat diet (HFD) along with low-level STZ injections (30 mg/kg) (Skovsø, 2014; Jin et al., 2023). At the conclusion of the experiment, the rats were euthanized with CO2, and femurs were harvested for subsequent evaluation.

To verify the impact of Met on DOP in rats, the animals were arbitrarily placed into four different groups: control group (Con, n = 6); DOP group (DOP, n = 6); Met treatment group (DOP + Met, n = 6), which received a daily dose of 200 mg/kg of Met via gavage (Jeyabalan et al., 2013; Loh et al., 2022); and Met + Compound C (Cc) group (DOP + Met + Cc, n = 6), which received daily doses of 200 mg/kg of Met via gavage and 0.2 mg/kg of Cc via intravenous injection (Kim et al., 2011). The Con and DOP groups received an equal volume of physiological saline through oral administration. Treatment with Met continued for 8 weeks. Following euthanasia via CO^2^ asphyxiation, the femurs were gathered for subsequent experiments. All surgical procedures and animal handling were authorized by the Animal Ethics Committee of the First Hospital of Jilin University (SYXK2022-0001).

### 2.3 Serum insulin and free fatty acids (FFAs)

Serum insulin concentration was evaluated utilizing an insulin ELISA kit (Solarbio, Shanghai, China) following the protocols. The concentration of FFAs in rat serum was determined following the instructions provided with the FFAs quantitative assay kit (Solarbio, Beijing, China).

### 2.4 Micro-CT analysis

Micro-CT scanning was conducted with equipment from PINGSENG Healthcare (Jiangsu, China) to analyze the microstructural characteristics of the rat femur. Regions of interest (ROI) were defined as 2 mm segments located below the growth plate at the distal femur and at the femoral diaphysis, where three-dimensional reconstruction and measurement were conducted.

### 2.5 Histomorphological and immunohistochemical (IHC) analyses

Hematoxylin and eosin (H&E) staining is employed for examining the pathological changes in bone tissue, including adipocytes and collagen fibers, and Masson’s trichrome staining allows for the examination of bone trabecular integrity and the thickness of collagen fibers. The femurs were fixed in 4% paraformaldehyde for 24 h, followed by decalcification in EDTA decalcification solution for 4 weeks. After decalcification, the femurs were dehydrated through a graded ethanol series and cleared in xylene. Subsequently, the samples were encapsulated in paraffin, and serial sections of 4 μm thickness were obtained along the coronal plane of the femur. H&E staining and Masson’s trichrome staining were conducted on the tissue sections, which were then mounted with neutral resin.

For IHC analysis, the 4 μm-thick tissue sections were first deparaffinized and subjected to antigen retrieval. Endogenous peroxidase activity was quenched in the sections. The sections were then incubated overnight at 4°C with a primary antibody against p-AMPK, GPX4, SLC7A11, followed by incubation with an HRP-conjugated secondary antibody at room temperature the next day. Finally, the sections were developed with DAB, counterstained with hematoxylin, dehydrated, and mounted with neutral resin for microscopic observation.

### 2.6 Cell culture

The osteoblast cell line MC3T3-E1 was obtained from Shanghai Enzyme Research Biotechnology Company. Cells were grown in a medium containing 10% FBS and 1% antibiotics in a 5% CO_2_ incubator at 37°C. MC3T3-E1 were pretreated with Met, Compound C (10 μmol/L) (Zhang et al., 2018; Wang X. et al., 2022) and AICAR(1 mM) (Yang et al., 2017; Lu et al., 2023) for 1 h prior to HGHF treatment, as required.

### 2.7 Cell viability analysis

To assess cell viability, a CCK-8 assay kit was purchased from NCM Biotech (Suzhou, China) and used following the guidelines. Absorbance readings were subsequently taken at a wavelength of 450 nm using a microplate reader from BMG LABtech (Germany).

### 2.8 Malondialdehyde and ferrous levels

Intracellular malondialdehyde (MDA) and ferrous levels are key indicators of ferroptosis. MDA levels were assessed with the MDA assay kit provided by Beyotime Biotechnology (Shanghai, China). The intracellular ferrous ion content was determined using a ferrous ion assay kit from Sigma-Aldrich (MO, United States), with strict adherence to the provided protocol.

### 2.9 Transmission electron microscopy

We utilized transmission electron microscopy (TEM) to examine the morphological changes in the mitochondria of the samples. The samples underwent a range of procedures, including fixation, dehydration, embedding, sectioning, and staining. Finally, imaging analysis of the samples was performed using a Hitachi 7800 TEM (Japan).

### 2.10 Alkaline phosphatase (ALP) analysis

MC3T3-E1 cells used for osteogenic differentiation were plated in a six-well plate and cultured under various treatment conditions. One portion of the samples was treated with the BCIP/NBT ALP color development kit (Beyotime, Shanghai, China) and photographed with a camera. Another portion of the samples was analyzed for ALP activity following the protocols provided by Beyotime (Shanghai, China), with absorbance taken at 405 nm using a microplate reader for semi-quantitative analysis.

### 2.11 Western Blot (WB) analysis

The extraction of total cellular protein and nuclear protein was carried out using kits from Beyotime Biotechnology. (Shanghai, China). Subsequently, gels were prepared using a PAGE rapid preparation kit (NCM Biotech, Suzhou, China) and subjected to electrophoresis. After electrophoresis, the gel was transferred onto a 0.45 μm PVDF membrane. This membrane was treated with 5% non-fat milk for 1 h and subsequently placed overnight at 4 °C with primary antibodies. Next, the PVDF membrane was washed five times with TBST and incubated with fluorescent secondary antibodies for 1 h. Finally, protein imaging was conducted utilizing FUSION Solo6S imaging system (VILBER, France).

### 2.12 Statistical analysis

All data are presented as mean ± standard deviation (SD). Each experiment was conducted a minimum of three times. Statistical analyses were performed utilizing GraphPad Prism 9 software (CA, United States). The Student’s t-test was applied for comparisons between two groups. When assessing multiple groups, one-way or two-way analysis of variance (ANOVA) with Tukey’s *post hoc* test was utilized. A p-value below 0.05 was deemed statistically significant.

## 3 Results

### 3.1 Ferroptosis in bone tissue was triggered by the diabetic microenvironment

**FIGURE 1:**
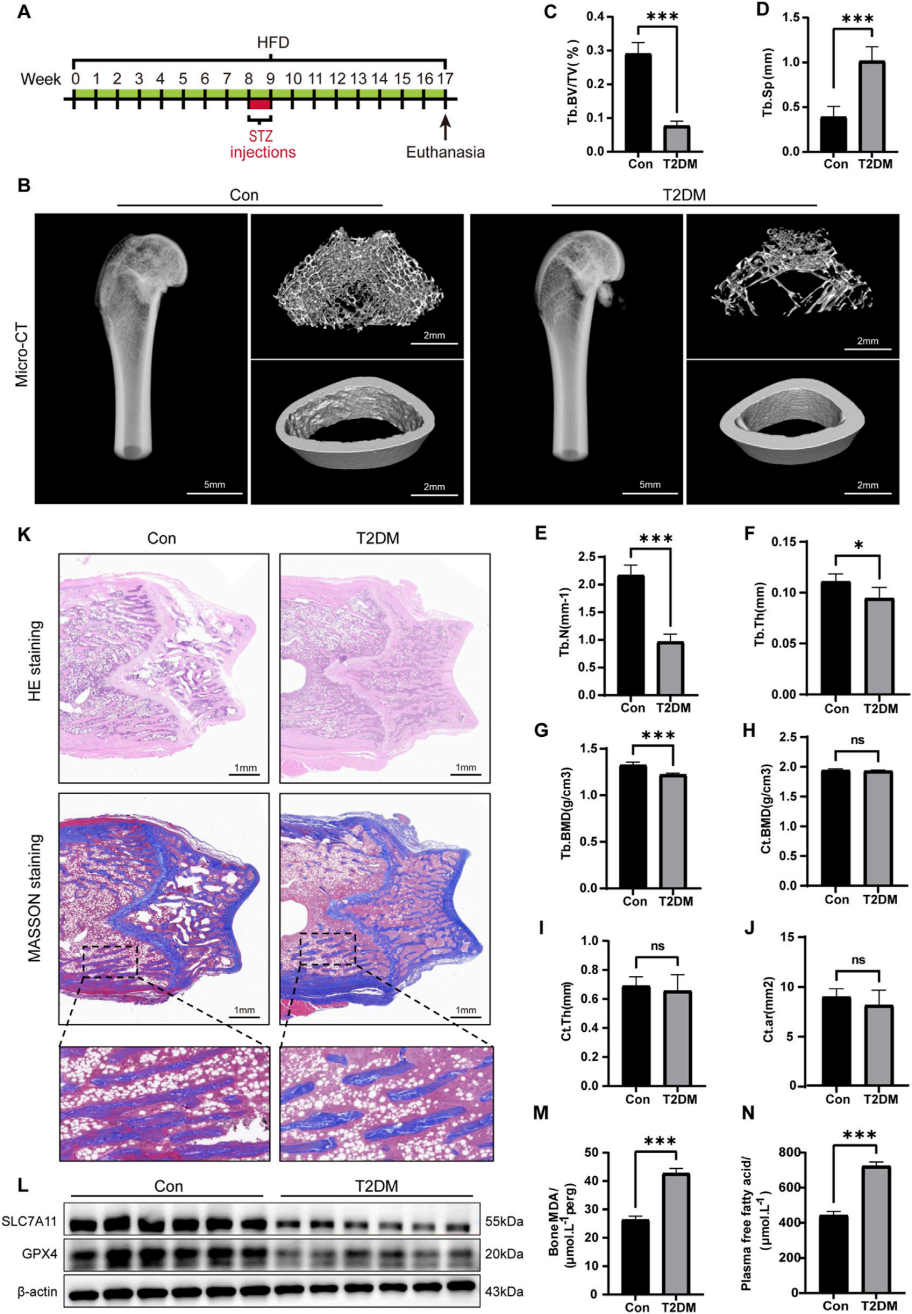
Ferroptosis in bone tissue induced by the diabetic microenvironment. **(A)** Illustrative diagram of the DOP model by administering a HFD alongside low-level STZ injections. **(B)** Representative micro-CT images depicting the microstructure of the femur. **(C–G)** Quantitative analysis of trabecular bone parameters. **(H–J)** Analysis of cortical bone parameters. **(K)** Representative images of H&E and Masson staining on the coronal section of the femoral metaphysis. **(L)** WB analysis of GPX4 and SLC7A11 in bone tissue. **(M)** MDA levels in bone tissue. **(N)** Serum FFA levels. ^*^ *P* < 0.05, ^**^ *P* < 0.01, ^***^ *P* < 0.001. Each group contained six rats.

To explore the potential mechanisms implicated in DOP, we initially developed a DOP rat model by administering an 8-week HFD alongside low-level STZ injections (30 mg/kg) administered once daily for three consecutive days (Figure 1A). Following STZ injections, the plasma glucose levels in the model group were markedly higher than those in the control group (Supplrmrntary Figure S1A). The increased fasting insulin levels further indicated the existence of insulin resistance in the model group (Supplrmrntary Figure S1B). These findings validated the effective creation of the T2DM rat model.

To assess the impact of T2DM on bone microstructure, we conducted micro-CT on the femurs of the rats. The results manifested that the trabecular bone structure at the distal femur of the T2DM group was significantly sparse compared to the Con (Figure 1B). Specifically, the trabecular bone parameters in the T2DM group, including Tb. BV/TV, Tb. N, Tb. BMD, and Tb. Th, were all significantly decreased, while Tb. Sp exhibited a notable increase (Figures 1C–G). However, there were no significant differences in the cortical bone parameters (Ct. BMD, Ct. Th, and Ct. Ar) between the Con and T2DM groups (Figures 1H–J). These findings suggested T2DM leads to a deterioration in bone density and microstructure of the trabecular bone at the distal femur, while the impact on cortical bone is not significant. We also conducted H&E and Masson staining on the trabecular bone at the distal femur to evaluate the morphological changes in bone tissue within the diabetic microenvironment. The results demonstrated the arrangement of trabecular bone was sparse, with widened spacing and thinning, along with fractures in the T2DM group. There was a notable increase in adipocytes within the trabecular cavities, and the collagen fibers in the local trabecular bone had disappeared (Figure 1K). These results collectively validated the effective creation of the DOP model.

Subsequently, we further investigated whether ferroptosis occurred in the bone tissue of DOP rats. We performed WB analysis on the expression of GPX4 and SLC7A11 (Figure 1L). The semi-quantitative analysis showed a significant decrease in the expression of GPX4 and SLC7A11 in the DOP rats (Supplrmrntary Figure S2). MDA is the terminal product of lipid peroxidation. Results showed the MDA levels in the DOP group were significantly elevated (Figure 1M), suggesting a high level of lipid peroxidation. Additionally, the serum levels of free fatty acids (FFAs) in the T2DM group were significantly increased (Figure 1N). This finding suggested elevated blood glucose and high FFA levels were major characteristics of the diabetic microenvironment. Palmitic acid (PA) is the most abundant saturated FFA and is known to be lipotoxic to osteoblasts (Al Saedi et al., 2020). Therefore, in subsequent *in vitro* cell experiments, we will simulate the type 2 diabetes microenvironment by exogenously adding glucose and PA (HGHF treatment). In summary, these findings collectively suggested the diabetic microenvironment induced ferroptosis in bone tissue *in vivo*.

### 3.2 Ferroptosis was induced by HGHF treatment in osteoblasts

**FIGURE 2:**
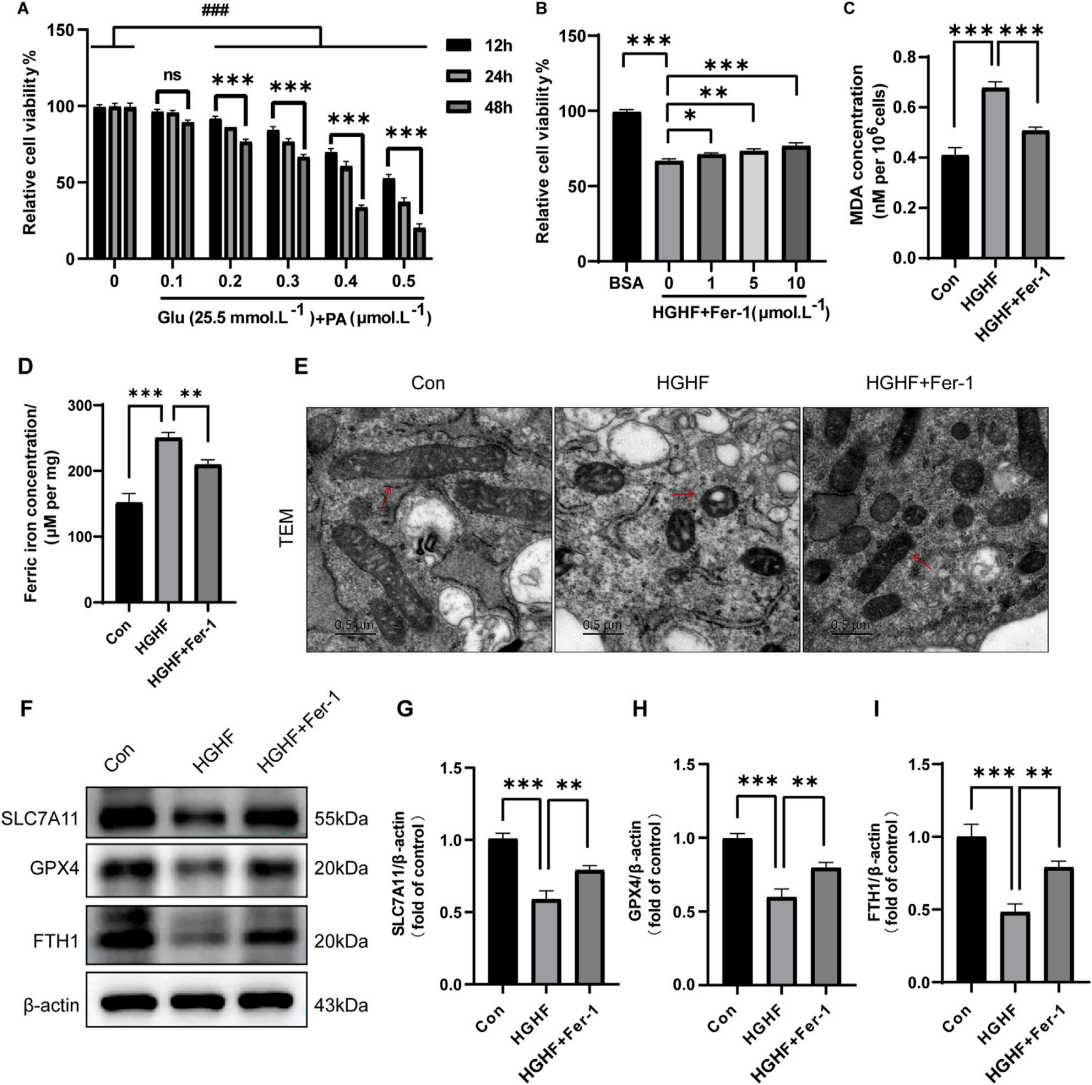
Ferroptosis was induced by HGHF treatment in osteoblasts. **(A)** Osteoblasts were cultured in a low glucose (5.5 mmol·L^−1^) environment or in the presence of various concentrations of PA along with 25.5 mmol·L^−1^ glucose, and cell viability was measured at different time points (n = 3). **(B)** After pre-treating osteoblasts with different concentrations of Fer-1, the cells were exposed to either BSA or HGHF for 48 h, followed by assessment of cell viability (n = 3). **(C)** Measurement of MDA levels in osteoblasts (n = 3). **(D)** Analysis of Fe^2+^ content in osteoblasts (n = 3). **(E)** Representative images of mitochondrial changes in osteoblasts observed via TEM (n = 3). **(F)** WB analysis of ferroptosis-related proteins (n = 3). **(G–I)** Semi-quantitative analysis of different ferroptosis markers (n = 3). ^*^ *P* < 0.05, ^**^ *P* < 0.01, ^***^ *P* < 0.001, ^###^ *P* < 0.001.

Building on previous studies and our findings on rat blood glucose levels, we applied a high concentration of glucose (HG, 25.5 mmol·L^−1^) to MC3T3-E1 cells to simulate the diabetic microenvironment, whereas the Con group was administered low glucose (LG, 5.5 mmol·L^−1^) (Ma et al., 2020). Additionally, MC3T3-E1 cells were exposed to a range of different concentrations of PA to determine the optimal concentration for inducing changes in osteoblasts (Figure 2A). Specifically, HGHF treatment markedly decreased osteoblast viability in a time-and dose-dependent manner, particularly at a PA concentration of 300 μmol L^−1^, where cell viability dropped to 66.7%. Therefore, this study established the experimental conditions of treating cells with 25.5 mM glucose and 300 μmol L^−1^ PA for 48 h to induce osteoblast death. Furthermore, to evaluate the rescue effect of different concentrations of Ferrostatin-1 (Fer-1, a ferroptosis inhibitor) on osteoblast viability, we introduced Fer-1 at concentrations ranging from 1 to 10 μmol L^−1^. The results indicated 10 μmol L^−1^ Fer-1 was the optimal concentration for following experiments (Figure 2B).

Ferroptosis is characterized by a range of morphological and biochemical changes, including alterations in mitochondrial morphology, iron overload, and lipid peroxidation. The results indicated HGHF treatment resulted in a notable elevation of MDA levels (Figure 2C), while Fer-1 treatment could partially restore this alteration. Moreover, the levels of ferrous iron (Fe^2+^) in each treatment group were analyzed using a Fe^2+^ detection kit. The findings indicated HGHF treatment significantly elevated the levels of Fe^2+^, with Fer-1 treatment showing some degree of improvement in this damage effect (Figure 2D).

Mitochondrial changes are regarded as key features of ferroptosis and are believed to act as amplifiers of this process. TEM observations indicated mitochondria in osteoblasts treated with HGHF exhibited shrinkage and outer membrane rupture, along with a deepening of mitochondrial color. In contrast, treatment with Fer-1 led to a partial recovery of mitochondrial structure (Figure 2E). Ultimately, WB analysis was performed to evaluate the expression of GPX4, SLC7A11, and FTH. The results demonstrated HGHF treatment markedly downregulated the expression of these proteins, while Fer-1 treatment was able to partially reverse this downregulation (Figures 2F–I). Together, these findings further confirm the occurrence of ferroptosis and elucidate its underlying mechanisms. In summary, ferroptosis was essential for HGHF-induced osteoblast ferroptosis *in vitro*.

### 3.3 Met inhibited HGHF-induced ferroptosis and improves osteogenic differentiation

**FIGURE 3:**
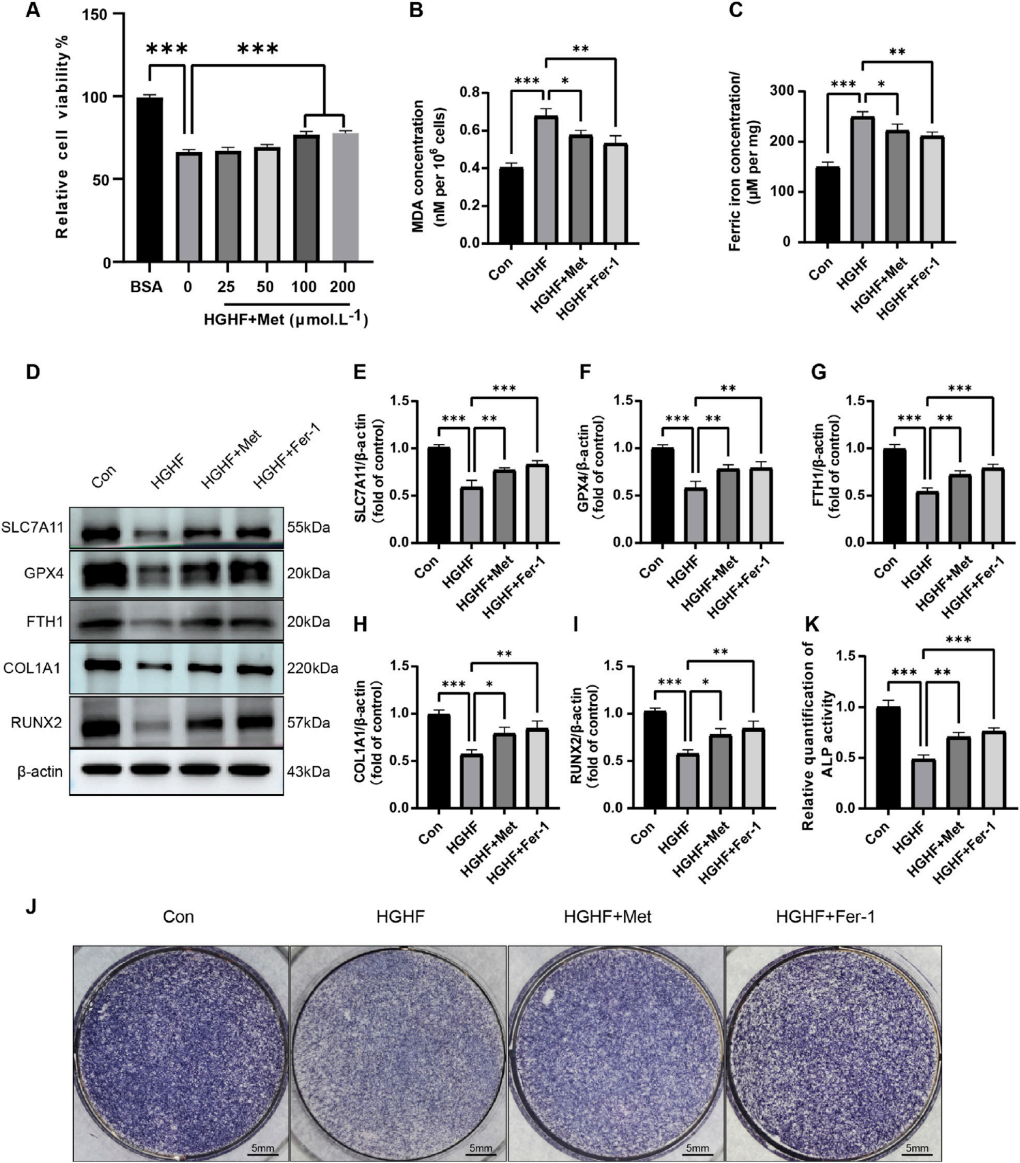
Met inhibited HGHF-induced Ferroptosis and improves osteogenic differentiation. **(A)** Changes in cell viability following 48 h of treatment with different concentrations of Met (n = 3). **(B)** Measurement of MDA levels in different treatment groups (n = 3). **(C)** Assessment of Fe^2+^ levels. **(D)** WB analysis of proteins associated with ferroptosis and osteogenic markers (n = 3). **(E–G)** Semi-quantitative analysis of ferroptosis-related proteins (n = 3). **(H, I)** Semi-quantitative analysis of osteogenic markers (n = 3). **(J)** Measurement of ALP in MC3T3-E1 cells on the seventh day of osteogenic induction (n = 3). **(K)** Semi-quantitative assessment of ALP (n = 3). ^*^ *P* < 0.05, ^**^ *P* < 0.01, ^***^ *P* < 0.001.

We exposed MC3T3-E1 cells affected by HGHF to varying concentrations of Met. The results indicated that treatment with 100 μmol L^−1^ Met significantly enhanced cell viability (Figure 3A). Therefore, we selected this concentration for subsequent cell experiments. Given that Met did not negatively impact cell viability or proliferation, we investigated its possible function in preventing HGHF-induced ferroptosis.

We first evaluated the effect of Met on intracellular MDA and ferrous ion levels under HGHF stimulation. HGHF treatment induced a marked elevation in MDA and ferrous ion levels, which was effectively decreased by post-treatment with Met or Fer-1, mitigating the associated oxidative damage (Figures 3B, C). Subsequent Western blot analysis revealed Met’s regulatory effects on key ferroptosis-related proteins. Met treatment partially reversed the downregulation of GPX4, SLC7A11, and FTH1 expression compared to the HGHF group (Figures 3D–G). These findings suggested Met effectively inhibited ferroptosis induced by HGHF in MC3T3-E1 cells.

Ferroptosis induced by HGHF hindered the osteogenic differentiation capacity of osteoblasts. Specifically, HGHF reduced the levels of osteogenic markers (COL1A1 and RUNX2), while both Met and Fer-1 effectively attenuated these HGHF-induced impairments (Figures 3D, H, I). Alkaline phosphatase (ALP), a crucial enzyme mediating bone mineralization, showed reduced activity following HGHF exposure. However, Met or Fer-1 treatment partially restored ALP activity, suggesting their protective effects on osteogenic function (Figures 3J, K). These results underscored the significant role of Met in counteracting the adverse impacts of HGHF on the osteogenic differentiation in MC3T3-E1 cells.

### 3.4 Met activated the AMPK/Nrf2 pathway to inhibit ferroptosis and osteogenic impairment in osteoblasts

**FIGURE 4:**
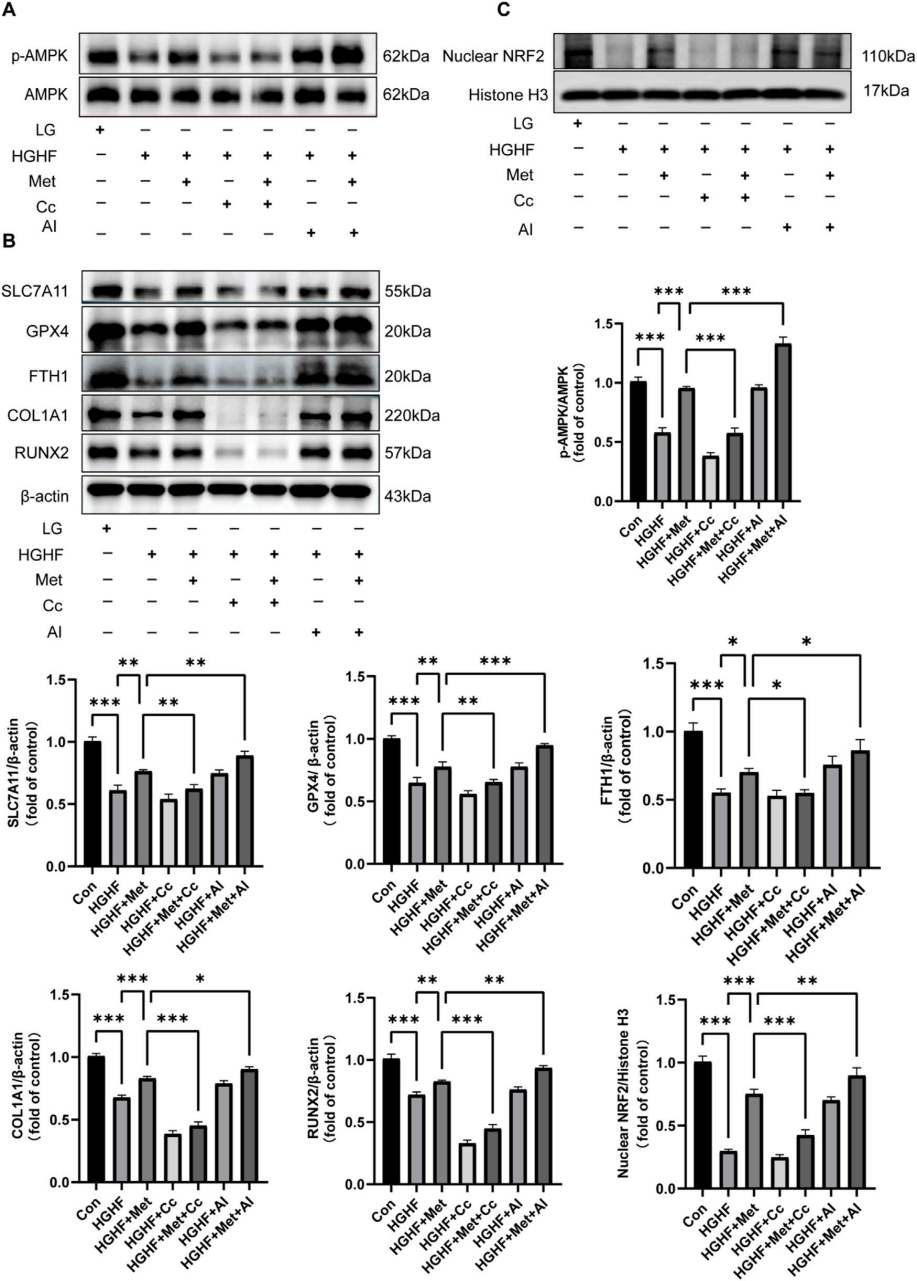
Met inhibited ferroptosis and osteogenic impairment via the AMPK/Nrf2 pathway. **(A)** WB analysis of p-AMPK and AMPK across various treatment groups (n = 3). **(B)** Analysis of changes in ferroptosis protective proteins and osteogenic markers (n = 3). **(C)** Expression of Nrf2 protein in the nucleus (n = 3). ^*^ *P* < 0.05, ^**^ *P* < 0.01, ^***^ *P* < 0.001.

AMPK is a crucial metabolic regulator that plays a significant role in cellular energy homeostasis (Madhavi et al., 2019). Given its potential involvement in suppressing ferroptosis, AMPK may serve as a therapeutic target for diseases associated with ferroptosis (Lee et al., 2020). To further investigate this mechanism, we conducted experiments using Compound C (Cc, an AMPK inhibitor), alongside AICAR (AI, an AMPK activator). WB analysis revealed Met significantly upregulated the expression of p-AMPK compared to the HGHF group (Figure 4A). As shown in Figure 4B, the upregulation of ferroptosis-protective proteins and osteogenic markers induced by Met was reversed following Cc intervention, whereas AI enhanced the protective effects of Met. These results demonstrated a critical connection between AMPK activation and ferroptosis.

Given the promoting effect of the AMPK pathway on Nrf2 nuclear accumulation and Nrf2’s antioxidant properties, we further investigated whether the inhibitory effect of Met on ferroptosis was associated with Nrf2 activation (Han et al., 2018). Our findings indicated Met treatment ameliorated the decline in nuclear Nrf2 protein expression induced by HGHF. The addition of Cc blocked the enhancing effect of Met on Nrf2 translocation, while AI strengthened this effect (Figure 4C). Collectively, these findings showed Met exerted a protective role against HGHF-induced ferroptosis and osteogenic differentiation in MC3T3 cells via the AMPK-dependent Nrf2 pathway.

### 3.5 Met inhibited ferroptosis and rescues DOP through the AMPK/Nrf2 pathway

**FIGURE 5:**
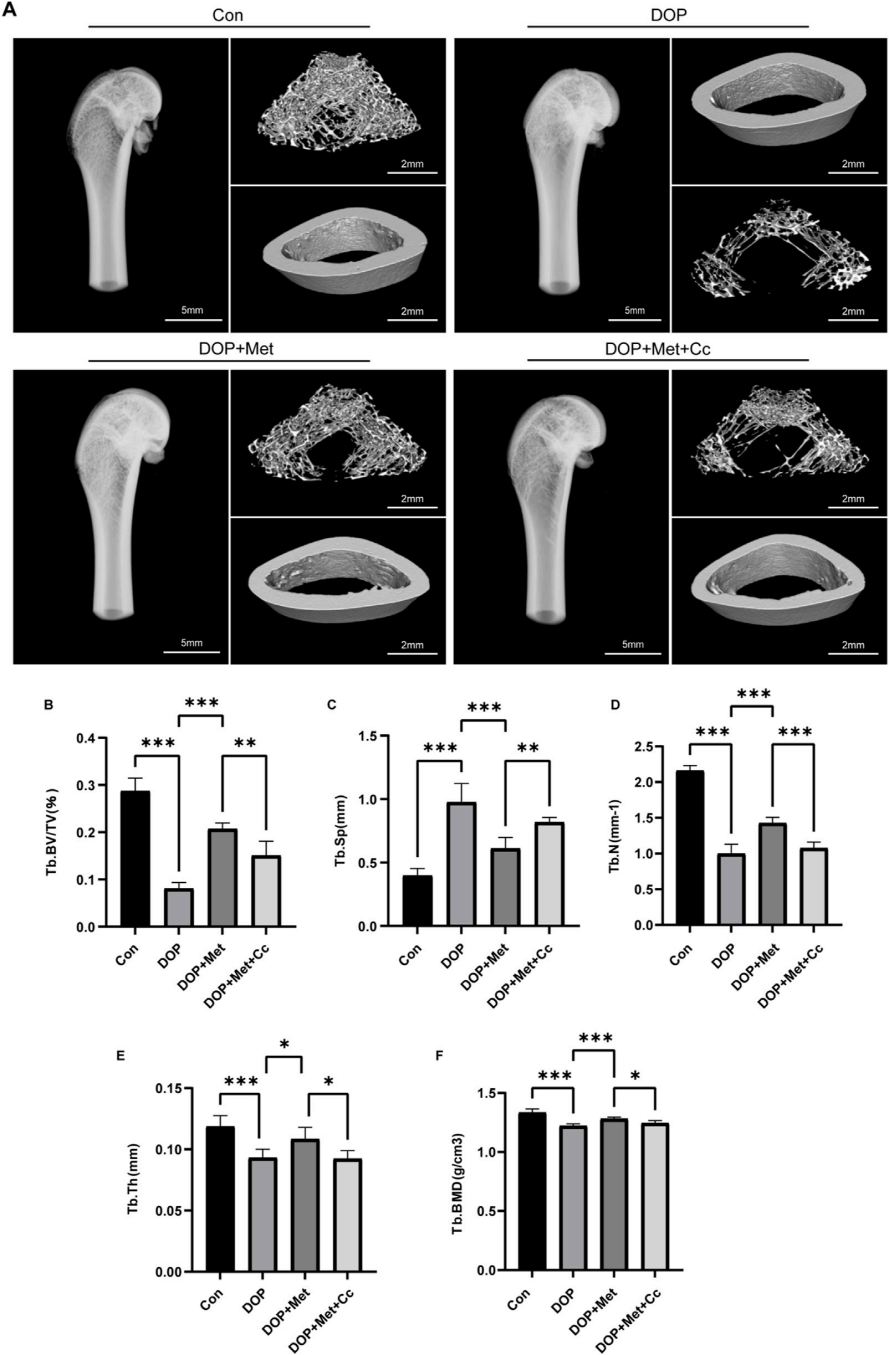
Met improved the microstructure of bone in the DOP model. **(A)** Representative micro-CT images from various treatment groups. **(B–F)** Quantitative analysis of trabecular bone parameters, including Tb. BV/TV, Tb. BMD, Tb. N, Tb. Th, and Tb. Sp. ^*^ *P* < 0.05, ^**^ *P* < 0.01, ^***^ *P* < 0.001. Each group contained six rats.

In the DOP rat model, we further investigated the effects of Met. Micro-CT revealed that an 8-week Met treatment significantly promoted the recovery of trabecular bone structure in the distal femur of DOP rats (Figure 5A). Specifically, parameters such as Tb. BV/TV, Tb. BMD, Tb. N, Tb. Th, and Tb. Sp showed significant improvement, although this enhancement was blocked by Cc intervention (Figures 5B–F).

**FIGURE 6:**
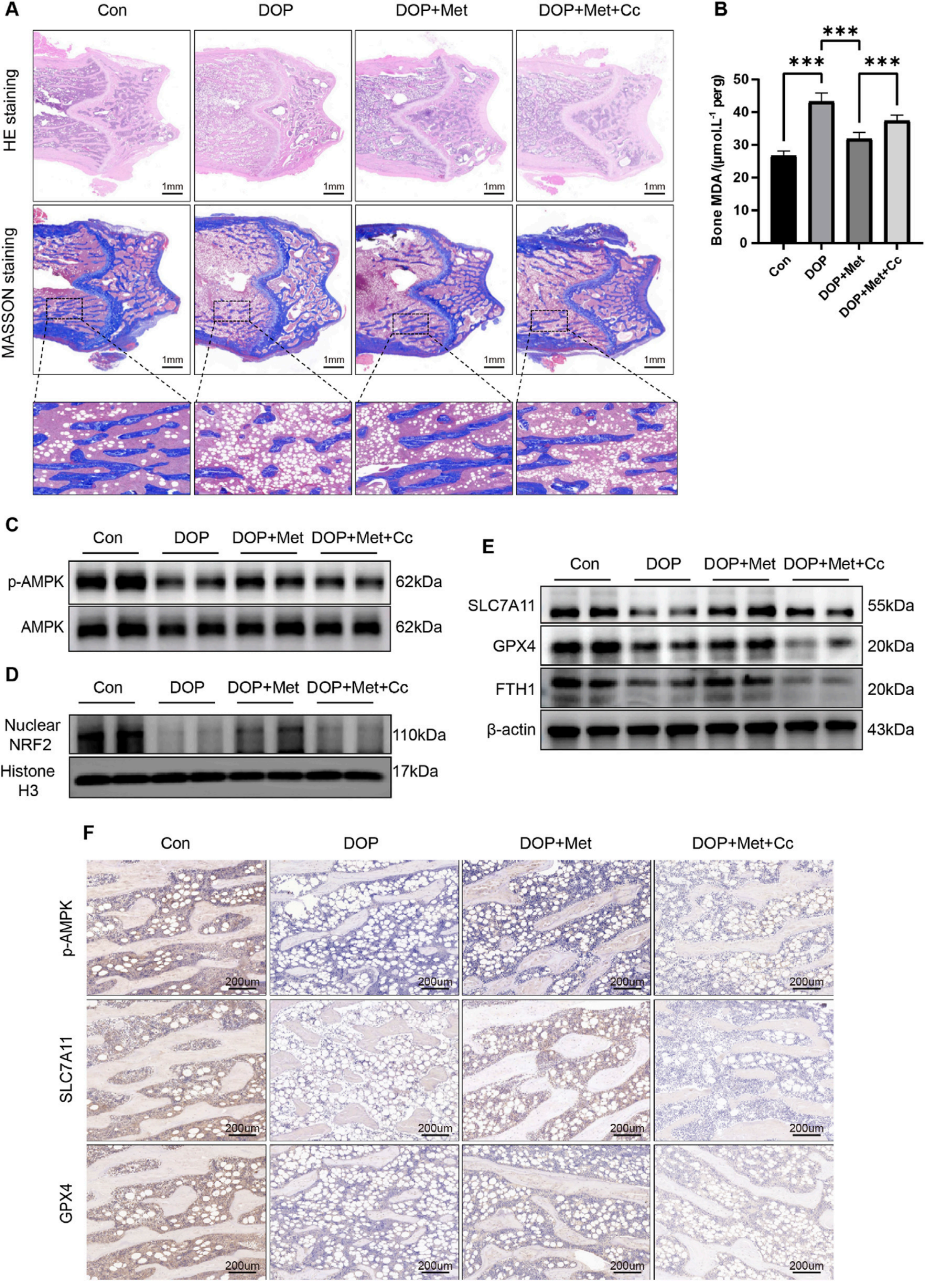
Met activated the AMPK/Nrf2 pathway to inhibit ferroptosis, rescuing DOP. **(A)** Representative coronal sections of the femoral metaphysis, displaying H&E and Masson staining images. **(B)** Measurement of MDA levels in bone tissue. **(C)** WB analysis of p-AMPK and AMPK expression in bone tissue. **(D)** Expression of Nrf2 protein in bone tissue. **(E)** WB analysis of ferroptosis-associated proteins in bone tissue. **(F)** IHC analysis of ferroptosis-associated proteins and p-AMPK in bone tissue. ^*^ *P* < 0.05, ^**^ *P* < 0.01, ^***^ *P* < 0.001. Each group contained six rats.

H&E and Masson staining were conducted on the distal femur. In comparison to the DOP group, the DOP + Met demonstrated enhanced trabecular bone density and a notable reduction in trabecular separation. In contrast, the Cc group diminished the protective effects of Met (Figure 6A). Met significantly reduced MDA levels in the DOP rats, while Cc partially attenuated this protective effect (Figure 6B). WB and IHC analyses demonstrated Met notably elevated the levels of Nrf2 and p-AMPK within the bone tissue of DOP rats, while the Cc group showed downregulation of these proteins (Figures 6C, D, F). Semi-quantitative analyses of these results are presented in Supplementary Figure S3A, B, 4A. Additionally, Met treatment enhanced the levels of ferroptosis-protective proteins like SLC7A11, GPX4, and FTH in DOP rats; however, these proteins were partially inhibited in the Met + Cc group (Figures 6E, F; Supplementary Figure S4B, C). Collectively, our findings demonstrated Met alleviated DOP through suppression of ferroptosis, highlighting its therapeutic potential for metabolic bone disorders.

## 4 Discussion

**FIGURE 7:**
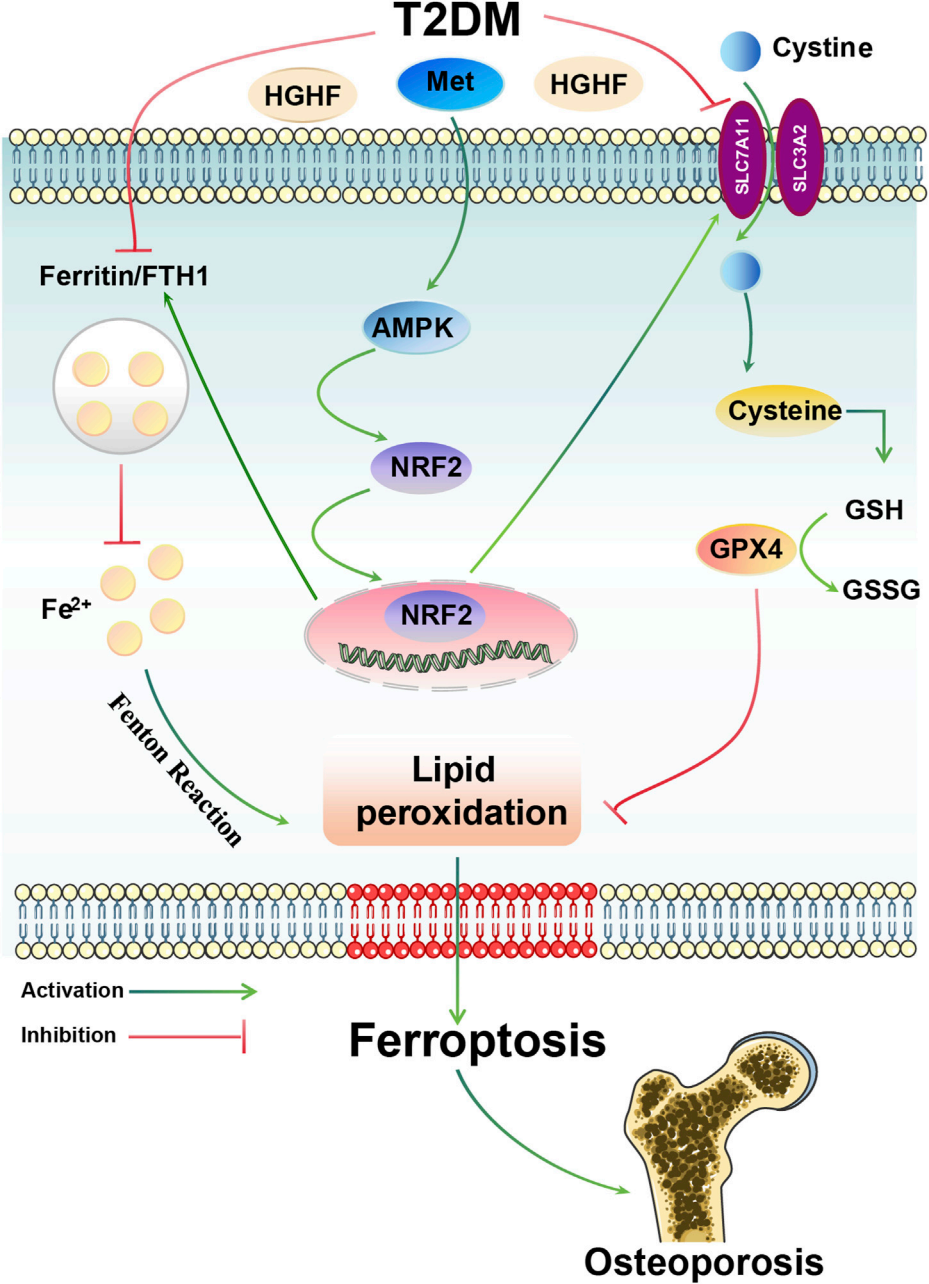
Overview of the mechanisms by which Met inhibits HGHF-induced ferroptosis and DOP. Met can attenuate HGHF-triggered ferroptosis, enhancing bone microarchitecture in DOP through the activation of the AMPK/Nrf2 signaling pathway.

In this study, we revealed ferroptosis is critical in the pathogenesis of DOP. Met, as a potential therapeutic intervention, effectively inhibited the process of lipid peroxidation and ferroptosis by activating the AMPK/Nrf2 signaling axis, thereby improving osteoporosis (Figure 7).

The concept of DOP was initially introduced in 1984 and is now widely acknowledged as a prevalent form of secondary osteoporosis (Takamoto and Kadowaki, 2004). In comparison to type 1 DOP, type 2 DOP exhibits more harsh deterioration in bone microstructure, marked by elevated cortical porosity and heightened bone fragility (2022). Recent studies further support the detrimental impacts of the diabetic microenvironment on bone strength and bone mass (Ma et al., 2020). Throughout the progression of DOP, excessive accumulation of glycotoxic metabolites occurs within bone tissue, leading to a reprogramming of osteoblast metabolism (Martiniakova et al., 2024). Additionally, the toxic effects of saturated FFAs on osteoblast function represent a critical factor in this context (Kim et al., 2008; Martiniakova et al., 2024).

In this research, we developed the type 2 DOP rat model using a HFD coupled with STZ injections. Micro-CT analysis revealed no significant changes in the cortical parameters of the rat femur (Ct. BMD, Ct. Th, and Ct. Ar), consistent with previous studies (Zhang et al., 2015). This lack of significant alteration may be attributed to the short time frame of the HFD modeling period, which could limit the impact on cortical bone remodeling. Additionally, we observed significant reductions in trabecular parameters, including Tb. BV/TV, Tb. N, Tb. BMD, and Tb. Th, while Tb. Sp showed a marked increase. This might be attributed to the greater impact of glycotoxicity on the actively remodeling trabecular regions of bone, a finding that aligns with previous conclusions (Botolin and McCabe, 2007; Motyl and McCabe, 2009). The deterioration of trabecular bone in DOP is associated with the suppression of osteogenesis and/or increased bone absorption. Numerous studies have demonstrated hyperglycemia and hyperlipidemia can directly suppress the osteogenic activity of osteoblasts, although the specific mechanisms remain unclear (Yamaguchi, 2010; Takanche et al., 2020). There is considerable divergence in the findings regarding the impact of the diabetic microenvironment on osteoclasts. A prolonged HFD alone does not significantly affect osteoclastogenesis; however, when combined with elevated blood glucose levels, there is a marked increase in both the quantity and activity of osteoclasts (An et al., 2019; Kim et al., 2021). However, it remains challenging to determine whether this osteoclast-promoting effect is directly caused by glycolipid metabolites or whether it results from the release of damage-associated factors within the diabetic microenvironment (Andreev et al., 2020).

Ferroptosis is a newly identified type of iron-dependent cell death, distinguished by excessive iron accumulation (Verma et al., 2020). Classical inducers of ferroptosis promote intracellular iron accumulation primarily by inhibiting antioxidant systems. The uptake of iron through transferrin receptor 1 increases free iron concentrations, while the storage of iron by FTH1 reduces free iron levels (Brown et al., 2019). Excessive free iron can lead to increased lipid peroxidation through Fenton reactions (Stockwell, 2022). In the process of ferroptosis, FTH1, GPX4 and SLC7A11 serve as key inhibitory factors. GPX4, as a central suppressor of ferroptosis, is an antioxidant enzyme that plays a crucial role in inhibiting lipid peroxidation (Mai et al., 2017; Zhang et al., 2021). SLC7A11 facilitates the uptake of extracellular cystine (the oxidized dimer form of cysteine), which is subsequently reduced to cysteine, participating in protein synthesis and other metabolic processes (Koppula et al., 2018). The correlation between ferroptosis and osteoporosis remains adequately underexplored, particularly osteoporosis induced by type 2 DOP. Studies have shown the Nrf2/HO-1 signaling pathway demonstrates potential in inhibiting high glucose-induced ferroptosis in type 2 DOP (Ma et al., 2020). Consistent with these findings, we observed that Fer-1 exhibited significant protective effects against ferroptosis triggered by HGHF conditions. Another important hallmark of ferroptosis is the alteration in mitochondrial morphology. Upon cellular stress, mitochondrial apoptosis typically initiates a cascade of cellular death. Given the crucial role of mitochondria in oxidative metabolism and apoptosis regulation, there exists a profound interplay between ferroptosis and mitochondrial function (Li et al., 2022). Our study revealed that osteoblasts treated with HGHF exhibited mitochondrial shrinkage and outer membrane rupture, alongside deepening mitochondrial coloration. In contrast, Met treatment partially restored mitochondrial structure. Furthermore, we observed that Met significantly mitigated lipid peroxidation induced by HGHF while markedly reducing the expression levels of SLC7A11, GPX4, and FTH1, suggesting Met exerts a beneficial effect on countering ferroptosis.

Met is a traditional and cost-effective first-line hypoglycemic agent (Shin et al., 2020). Numerous studies have demonstrated that Met can improve diabetes and its complications (Hu et al., 2020; Ala and Ala, 2021). Met significantly enhances the phosphorylation levels of AMPK in osteoblasts, which aligns with the findings of this study (LaMoia and Shulman, 2021). Additionally, research suggests that Met-induced AMPK activation may serve as an effective therapeutic strategy to prevent osteoblast apoptosis (Jang et al., 2011). However, the particular function of Met regarding type 2 DOP warrants further investigation. AMPK, as one of the action targets of Met, is an enzyme that is widely expressed in various tissues, including the heart, kidneys, liver, brain, bone, and skeletal muscle (Lee et al., 2020). As a sensor of cellular energy status, dysfunction of AMPK can lead to a variety of human diseases, particularly metabolic disorders (Peng et al., 2024). Recent studies have indicated that cancer cells exhibit resistance to ferroptosis due to high levels of AMPK activity, while cancer cells with inactivated AMPK are more prone to ferroptosis (Yang et al., 2021). However, whether Met exerts its inhibitory effect on HGHF-induced ferroptosis through AMPK activation has yet to be investigated. To confirm this hypothesis, we evaluated the impact of AMPK phosphorylation on ferroptosis. We observed the expression of p-AMPK was observably reduced in MC3T3-E1 cells treated with HGHF and in bone tissue from DOP rats, a condition that was partially ameliorated by Met treatment. Furthermore, co-treatment with Met and the AMPK activator AI enhanced the antioxidant capacity against ferroptosis in MC3T3-E1 cells exposed to HGHF. In contrast, the application of the AMPK inhibitor Cc under HGHF conditions diminished these protective benefits and heightened the susceptibility of MC3T3-E1 cells to ferroptosis. Collectively, these findings demonstrated Met ameliorated DOP through ferroptosis inhibition. Unlike conventional anti-osteoporotic agents such as bisphosphonates, which primarily act by suppressing osteoclastic bone resorption (Coe et al., 2015), Met exhibits dual therapeutic benefits: glycemic control and osteogenic enhancement. Although bisphosphonates increase bone mineral density in elderly women with T2DM, they concomitantly reduce osteogenic marker, potentially leading to secondary suppression of bone formation (Keegan et al., 2004; Gangoiti et al., 2008).

Nrf2 acts as a vital transcription factor responsible for maintaining redox balance within cells. Under normal physiological conditions, Nrf2 is highly unstable in the cytoplasm, where it is rapidly ubiquitinated and degraded via the proteasome pathway (Adelusi et al., 2020). In response to stress, Nrf2 can move into the nucleus and stimulate the transcription of genes associated with antioxidant response elements (Zhao et al., 2022). However, following prolonged oxidative stress, Nrf2 expression levels gradually decrease, exacerbating oxidative damage (Mathur et al., 2018; Wang et al., 2020). Activation of Nrf2 can upregulate the expression of various target genes, including GPX4, FTH1, and SLC7A11 (Dodson et al., 2019a). The Nrf2/SLC7A11/GPX4 axis has been shown to inhibit ferroptosis and oxidative stress induced by cerebral ischemia-reperfusion, thus exerting neuroprotective effects (Yuan et al., 2021). Research has indicated that activation of the AMPK signaling pathway can promote the accumulation of Nrf2 in the nucleus, enhancing its antioxidant activity in models of renal ischemia-reperfusion injury and diabetic cardiomyopathy (Wang X. et al., 2022; Kuang et al., 2023). Nevertheless, the contribution of the AMPK/Nrf2 pathway to the effects of Met on ferroptosis in DOP remains ambiguous. Our study found HGHF treatment reduced the antioxidant defense capacity of Nrf2 in MC3T3-E1 cells and diminished its nuclear translocation. Met activated AMPK and promoted the movement of Nrf2 into the nucleus, leading to upregulation of protein expression for FTH1, SLC7A11, and GPX4, which alleviated lipid peroxidation and the following ferroptosis. Furthermore, Met treatment enhanced the ability for osteogenic differentiation in MC3T3-E1 cells. *In vivo* experiments further demonstrated that Met administration reduced MDA levels in the femur and elevated the levels of GPX4, SLC7A11, and FTH1 in the distal femur, ultimately improving bone microstructure.

Our study has several limitations. First, while clinical bone specimens from DOP patients are challenging to obtain, utilizing primary human osteoblasts could provide additional validation of our findings. Second, the absence of genetic models targeting key ferroptosis regulators (such as GPX4) limits our mechanistic understanding of Met’s effects on ferroptosis pathways. Finally, our investigation focused on the 8-week therapeutic window, leaving the long-term efficacy and dose-dependent effects of Met in DOP management an important area for future research.

## 5 Conclusion

This research demonstrates the important role of ferroptosis in the development of DOP. In the DOP microenvironment, Met prevents ferroptosis and enhances osteogenic differentiation by activating the AMPK/Nrf2 pathway. Our results emphasize the promise of Met as a therapeutic agent to inhibit ferroptosis and highlight its prospective value in osteoporosis treatment.

## Data Availability

The original contributions presented in the study are included in the article/Supplementary Material, further inquiries can be directed to the corresponding author.

## Abbreviations

Met, metformin; DOP, diabetic osteoporosis; MDA, malondialdehyde; AMPK, AMP-activated protein kinase; FTH1, ferritin heavy chain 1; GPx4, glutathione peroxidase 4; Nrf2, nuclear factor erythroid 2-related factor 2; SLC7A11, solute carrier family 7, member 11; ALP, alkaline phosphatase; FFAs, free fatty acids; Tb.BV/TV, trabecular bone volume/tissue volume; Tb.N, trabecular number; Tb.Th, trabecular thickness; Tb.Sp, trabecular separation; Tb.BMD, trabecular bone mineral density; Ct.BMD, cortical bone mineral density; Ct.Th, cortical bone thickness; Ct.Ar, cortical bone area.

## References

[B1] Adelusi T. I. Du L. Hao M. Zhou X. Xuan Q. Apu C. (2020). Keap1/Nrf2/ARE signaling unfolds therapeutic targets for redox imbalanced-mediated diseases and diabetic nephropathy. Biomed. Pharmacother. 123, 109732. 10.1016/j.biopha.2019.109732 31945695

[B2] Ala M. Ala M. (2021). Metformin for cardiovascular protection, inflammatory bowel disease, osteoporosis, periodontitis, polycystic ovarian syndrome, neurodegeneration, cancer, inflammation and senescence: what is next? ACS Pharmacol. Transl. Sci. 4 (6), 1747–1770. 10.1021/acsptsci.1c00167 34927008 PMC8669709

[B3] Al Saedi A. Myers D. E. Stupka N. Duque G. (2020). 1,25(OH)(2)D(3) ameliorates palmitate-induced lipotoxicity in human primary osteoblasts leading to improved viability and function. Bone 141, 115672. 10.1016/j.bone.2020.115672 33011427

[B4] American Diabetes Association Professional Practice Committee (2022). 2. Classification and diagnosis of diabetes: standards of medical care in diabetes-2022. Diabetes Care 45 (Suppl. 1), S17–s38. 10.2337/dc22-S002 34964875

[B5] An Y. Zhang H. Wang C. Jiao F. Xu H. Wang X. (2019). Activation of ROS/MAPKs/NF-κB/NLRP3 and inhibition of efferocytosis in osteoclast-mediated diabetic osteoporosis. Faseb J. 33 (11), 12515–12527. 10.1096/fj.201802805RR 31461386 PMC6902677

[B6] Andreev D. Liu M. Weidner D. Kachler K. Faas M. Grüneboom A. (2020). Osteocyte necrosis triggers osteoclast-mediated bone loss through macrophage-inducible C-type lectin. J. Clin. Invest 130 (9), 4811–4830. 10.1172/jci134214 32773408 PMC7456234

[B7] Bootman M. D. Chehab T. Bultynck G. Parys J. B. Rietdorf K. (2018). The regulation of autophagy by calcium signals: do we have a consensus? Cell Calcium 70, 32–46. 10.1016/j.ceca.2017.08.005 28847414

[B8] Botolin S. McCabe L. R. (2007). Bone loss and increased bone adiposity in spontaneous and pharmacologically induced diabetic mice. Endocrinology 148 (1), 198–205. 10.1210/en.2006-1006 17053023

[B9] Brown C. W. Amante J. J. Chhoy P. Elaimy A. L. Liu H. Zhu L. J. (2019). Prominin2 drives ferroptosis resistance by stimulating iron export. Dev. Cell 51 (5), 575–586.e4. 10.1016/j.devcel.2019.10.007 31735663 PMC8316835

[B10] Coe L. M. Tekalur S. A. Shu Y. Baumann M. J. McCabe L. R. (2015). Bisphosphonate treatment of type I diabetic mice prevents early bone loss but accentuates suppression of bone formation. J. Cell Physiol. 230 (8), 1944–1953. 10.1002/jcp.24929 25641511 PMC4724196

[B11] Dodson M. Castro-Portuguez R. Zhang D. D. (2019a). NRF2 plays a critical role in mitigating lipid peroxidation and ferroptosis. Redox Biol. 23, 101107. 10.1016/j.redox.2019.101107 30692038 PMC6859567

[B12] Dodson M. de la Vega M. R. Cholanians A. B. Schmidlin C. J. Chapman E. Zhang D. D. (2019b). Modulating NRF2 in disease: timing is everything. Annu. Rev. Pharmacol. Toxicol. 59, 555–575. 10.1146/annurev-pharmtox-010818-021856 30256716 PMC6538038

[B13] Dong H. Xia Y. Jin S. Xue C. Wang Y. Hu R. (2021). Nrf2 attenuates ferroptosis-mediated IIR-ALI by modulating TERT and SLC7A11. Cell Death Dis. 12 (11), 1027. 10.1038/s41419-021-04307-1 34716298 PMC8556385

[B14] Gangoiti M. V. Cortizo A. M. Arnol V. Felice J. I. McCarthy A. D. (2008). Opposing effects of bisphosphonates and advanced glycation end-products on osteoblastic cells. Eur. J. Pharmacol. 600 (1-3), 140–147. 10.1016/j.ejphar.2008.10.031 18973752

[B15] Gao Z. Chen Z. Xiong Z. Liu X. (2022). Ferroptosis - a new target of osteoporosis. Exp. Gerontol. 165, 111836. 10.1016/j.exger.2022.111836 35598699

[B16] Han L. Yang Q. Ma W. Li J. Qu L. Wang M. (2018). Protocatechuic acid ameliorated palmitic-acid-induced oxidative damage in endothelial cells through activating endogenous antioxidant enzymes via an adenosine-monophosphate-activated-protein-kinase-dependent pathway. J. Agric. Food Chem. 66 (40), 10400–10409. 10.1021/acs.jafc.8b03414 30220205

[B17] Hu Y. Lei M. Ke G. Huang X. Peng X. Zhong L. (2020). Metformin use and risk of all-cause mortality and cardiovascular events in patients with chronic kidney disease-A systematic review and meta-analysis. Front. Endocrinol. (Lausanne) 11, 559446. 10.3389/fendo.2020.559446 33117278 PMC7575818

[B18] Jang W. G. Kim E. J. Lee K. N. Son H. J. Koh J. T. (2011). AMP-activated protein kinase (AMPK) positively regulates osteoblast differentiation via induction of Dlx5-dependent Runx2 expression in MC3T3E1 cells. Biochem. Biophys. Res. Commun. 404 (4), 1004–1009. 10.1016/j.bbrc.2010.12.099 21187071

[B19] Jeyabalan J. Viollet B. Smitham P. Ellis S. A. Zaman G. Bardin C. (2013). The anti-diabetic drug metformin does not affect bone mass *in vivo* or fracture healing. Osteoporos. Int. 24 (10), 2659–2670. 10.1007/s00198-013-2371-0 23644877 PMC3777188

[B20] Jin C. Tan K. Yao Z. Lin B. H. Zhang D. P. Chen W. K. (2023). A novel anti-osteoporosis mechanism of VK2: interfering with ferroptosis via AMPK/SIRT1 pathway in type 2 diabetic osteoporosis. J. Agric. Food Chem. 71 (6), 2745–2761. 10.1021/acs.jafc.2c05632 36719855

[B21] Johnell O. Kanis J. A. (2006). An estimate of the worldwide prevalence and disability associated with osteoporotic fractures. Osteoporos. Int. 17 (12), 1726–1733. 10.1007/s00198-006-0172-4 16983459

[B22] Keegan T. H. Schwartz A. V. Bauer D. C. Sellmeyer D. E. Kelsey J. L. fracture intervention trial (2004). Effect of alendronate on bone mineral density and biochemical markers of bone turnover in type 2 diabetic women: the fracture intervention trial. Diabetes Care 27 (7), 1547–1553. 10.2337/diacare.27.7.1547 15220226

[B23] Kim J. E. Ahn M. W. Baek S. H. Lee I. K. Kim Y. W. Kim J. Y. (2008). AMPK activator, AICAR, inhibits palmitate-induced apoptosis in osteoblast. Bone 43 (2), 394–404. 10.1016/j.bone.2008.03.021 18502715

[B24] Kim S. Henneicke H. Cavanagh L. L. Macfarlane E. Thai L. J. Foong D. (2021). Osteoblastic glucocorticoid signaling exacerbates high-fat-diet- induced bone loss and obesity. Bone Res. 9 (1), 40. 10.1038/s41413-021-00159-9 34465731 PMC8408138

[B25] Kim Y. M. Kim M. Y. Kim H. J. Roh G. S. Ko G. H. Seo H. G. (2011). Compound C independent of AMPK inhibits ICAM-1 and VCAM-1 expression in inflammatory stimulants-activated endothelial cells *in vitro* and *in vivo* . Atherosclerosis 219 (1), 57–64. 10.1016/j.atherosclerosis.2011.06.043 21764059

[B26] Koppula P. Zhang Y. Zhuang L. Gan B. (2018). Amino acid transporter SLC7A11/xCT at the crossroads of regulating redox homeostasis and nutrient dependency of cancer. Cancer Commun. (Lond) 38 (1), 12. 10.1186/s40880-018-0288-x 29764521 PMC5993148

[B27] Kuang B. C. Wang Z. H. Hou S. H. Zhang J. Wang M. Q. Zhang J. S. (2023). Methyl eugenol protects the kidney from oxidative damage in mice by blocking the Nrf2 nuclear export signal through activation of the AMPK/GSK3β axis. Acta Pharmacol. Sin. 44 (2), 367–380. 10.1038/s41401-022-00942-2 35794373 PMC9889399

[B28] LaMoia T. E. Shulman G. I. (2021). Cellular and molecular mechanisms of metformin action. Endocr. Rev. 42 (1), 77–96. 10.1210/endrev/bnaa023 32897388 PMC7846086

[B29] Lee H. Zandkarimi F. Zhang Y. Meena J. K. Kim J. Zhuang L. (2020). Energy-stress-mediated AMPK activation inhibits ferroptosis. Nat. Cell Biol. 22 (2), 225–234. 10.1038/s41556-020-0461-8 32029897 PMC7008777

[B30] Li J. Wu F. Xiao X. Su L. Guo X. Yao J. (2022). A novel ferroptosis-related gene signature to predict overall survival in patients with osteosarcoma. Am. J. Transl. Res. 14 (9), 6082–6094. 36247280 PMC9556449

[B31] Liu P. Wang W. Li Z. Li Y. Yu X. Tu J. (2022). Ferroptosis: a new regulatory mechanism in osteoporosis. Oxid. Med. Cell Longev. 2022, 2634431. 10.1155/2022/2634431 35082963 PMC8786466

[B32] Loh D. K. W. Kadirvelu A. Pamidi N. (2022). Effects of metformin on bone mineral density and adiposity-associated pathways in animal models with type 2 diabetes mellitus: a systematic review. J. Clin. Med. 11 (14), 4193. 10.3390/jcm11144193 35887957 PMC9323116

[B33] Lu Q. Yang L. Xiao J. J. Liu Q. Ni L. Hu J. W. (2023). Empagliflozin attenuates the renal tubular ferroptosis in diabetic kidney disease through AMPK/NRF2 pathway. Free Radic. Biol. Med. 195, 89–102. 10.1016/j.freeradbiomed.2022.12.088 36581059

[B34] Lu Y. Yuan T. Min X. Yuan Z. Cai Z. (2021). AMPK: potential therapeutic target for vascular calcification. Front. Cardiovasc Med. 8, 670222. 10.3389/fcvm.2021.670222 34046440 PMC8144331

[B35] Lv Z. Guo Y. (2020). Metformin and its benefits for various diseases. Front. Endocrinol. (Lausanne) 11, 191. 10.3389/fendo.2020.00191 32425881 PMC7212476

[B36] Ma H. Wang X. Zhang W. Li H. Zhao W. Sun J. (2020). Melatonin suppresses ferroptosis induced by high glucose via activation of the Nrf2/HO-1 signaling pathway in type 2 diabetic osteoporosis. Oxid. Med. Cell Longev. 2020, 9067610. 10.1155/2020/9067610 33343809 PMC7732386

[B37] Madhavi Y. V. Gaikwad N. Yerra V. G. Kalvala A. K. Nanduri S. Kumar A. (2019). Targeting AMPK in diabetes and diabetic complications: energy homeostasis, autophagy and mitochondrial health. Curr. Med. Chem. 26 (27), 5207–5229. 10.2174/0929867325666180406120051 29623826

[B38] Mai T. T. Hamaï A. Hienzsch A. Cañeque T. Müller S. Wicinski J. (2017). Salinomycin kills cancer stem cells by sequestering iron in lysosomes. Nat. Chem. 9 (10), 1025–1033. 10.1038/nchem.2778 28937680 PMC5890907

[B39] Martiniakova M. Biro R. Penzes N. Sarocka A. Kovacova V. Mondockova V. (2024). Links among obesity, type 2 diabetes mellitus, and osteoporosis: bone as a target. Int. J. Mol. Sci. 25 (9), 4827. 10.3390/ijms25094827 38732046 PMC11084398

[B40] Mathur A. Pandey V. K. Kakkar P. (2018). Activation of GSK3β/β-TrCP axis via PHLPP1 exacerbates Nrf2 degradation leading to impairment in cell survival pathway during diabetic nephropathy. Free Radic. Biol. Med. 120, 414–424. 10.1016/j.freeradbiomed.2018.04.550 29655866

[B41] Motyl K. McCabe L. R. (2009). Streptozotocin, type I diabetes severity and bone. Biol. Proced. Online 11, 296–315. 10.1007/s12575-009-9000-5 19495918 PMC3055251

[B42] Napoli N. Chandran M. Pierroz D. D. Abrahamsen B. Schwartz A. V. Ferrari S. L. (2017). Mechanisms of diabetes mellitus-induced bone fragility. Nat. Rev. Endocrinol. 13 (4), 208–219. 10.1038/nrendo.2016.153 27658727

[B43] Peng Y. Qi Z. Xu Y. Yang X. Cui Y. Sun Q. (2024). AMPK and metabolic disorders: the opposite roles of dietary bioactive components and food contaminants. Food Chem. 437 (Pt 1), 137784. 10.1016/j.foodchem.2023.137784 37897819

[B44] Pollak M. (2017). The effects of metformin on gut microbiota and the immune system as research frontiers. Diabetologia 60 (9), 1662–1667. 10.1007/s00125-017-4352-x 28770326

[B45] Shin J. I. Sang Y. Chang A. R. Dunning S. C. Coresh J. Inker L. A. (2020). The FDA metformin label change and racial and sex disparities in metformin prescription among patients with CKD. J. Am. Soc. Nephrol. 31 (8), 1847–1858. 10.1681/asn.2019101119 32660971 PMC7460896

[B46] Skovsø S. (2014). Modeling type 2 diabetes in rats using high fat diet and streptozotocin. J. Diabetes Investig. 5 (4), 349–358. 10.1111/jdi.12235 PMC4210077 25411593

[B47] Stockwell B. R. (2022). Ferroptosis turns 10: emerging mechanisms, physiological functions, and therapeutic applications. Cell 185 (14), 2401–2421. 10.1016/j.cell.2022.06.003 35803244 PMC9273022

[B48] Stockwell B. R. Friedmann Angeli J. P. Bayir H. Bush A. I. Conrad M. Dixon S. J. (2017). Ferroptosis: a regulated cell death nexus linking metabolism, redox biology, and disease. Cell 171 (2), 273–285. 10.1016/j.cell.2017.09.021 28985560 PMC5685180

[B49] Sun H. Saeedi P. Karuranga S. Pinkepank M. Ogurtsova K. Duncan B. B. (2022). IDF Diabetes Atlas: global, regional and country-level diabetes prevalence estimates for 2021 and projections for 2045. Diabetes Res. Clin. Pract. 183, 109119. 10.1016/j.diabres.2021.109119 34879977 PMC11057359

[B50] Takamoto I. Kadowaki T. (2004). Diabetes and osteoporosis. Clin. Calcium 14 (2), 255–261. 15576981

[B51] Takanche J. S. Kim J. E. Han S. H. Yi H. K. (2020). Effect of gomisin A on osteoblast differentiation in high glucose-mediated oxidative stress. Phytomedicine 66, 153107. 10.1016/j.phymed.2019.153107 31790903

[B52] Verma N. Vinik Y. Saroha A. Nair N. U. Ruppin E. Mills G. (2020). Synthetic lethal combination targeting BET uncovered intrinsic susceptibility of TNBC to ferroptosis. Sci. Adv. 6 (34), eaba8968. 10.1126/sciadv.aba8968 32937365 PMC7442484

[B53] Wang S. Nie P. Lu X. Li C. Dong X. Yang F. (2020). Nrf2 participates in the anti-apoptotic role of zinc in Type 2 diabetic nephropathy through Wnt/β-catenin signaling pathway. J. Nutr. Biochem. 84, 108451. 10.1016/j.jnutbio.2020.108451 32795642

[B54] Wang X. Chen X. Zhou W. Men H. Bao T. Sun Y. (2022a). Ferroptosis is essential for diabetic cardiomyopathy and is prevented by sulforaphane via AMPK/NRF2 pathways. Acta Pharm. Sin. B 12 (2), 708–722. 10.1016/j.apsb.2021.10.005 35256941 PMC8897044

[B55] Wang Y. Yan S. Liu X. Deng F. Wang P. Yang L. (2022b). PRMT4 promotes ferroptosis to aggravate doxorubicin-induced cardiomyopathy via inhibition of the Nrf2/GPX4 pathway. Cell Death Differ. 29 (10), 1982–1995. 10.1038/s41418-022-00990-5 35383293 PMC9525272

[B56] Xian H. Liu Y. Rundberg Nilsson A. Gatchalian R. Crother T. R. Tourtellotte W. G. (2021). Metformin inhibition of mitochondrial ATP and DNA synthesis abrogates NLRP3 inflammasome activation and pulmonary inflammation. Immunity 54 (7), 1463–1477.e11. 10.1016/j.immuni.2021.05.004 34115964 PMC8189765

[B57] Yamaguchi T. (2010). Bone fragility in type 2 diabetes mellitus. World J. Orthop. 1 (1), 3–9. 10.5312/wjo.v1.i1.3 22474621 PMC3302026

[B58] Yang J. Zhou Y. Xie S. Wang J. Li Z. Chen L. (2021). Metformin induces Ferroptosis by inhibiting UFMylation of SLC7A11 in breast cancer. J. Exp. Clin. Cancer Res. 40 (1), 206. 10.1186/s13046-021-02012-7 34162423 PMC8223374

[B59] Yang S. Zhao L. Han Y. Liu Y. Chen C. Zhan M. (2017). Probucol ameliorates renal injury in diabetic nephropathy by inhibiting the expression of the redox enzyme p66Shc. Redox Biol. 13, 482–497. 10.1016/j.redox.2017.07.002 28728079 PMC5514499

[B60] Yuan Y. Zhai Y. Chen J. Xu X. Wang H. (2021). Kaempferol ameliorates oxygen-glucose deprivation/reoxygenation-induced neuronal ferroptosis by activating nrf2/slc7a11/GPX4 Axis. Biomolecules 11 (7), 923. 10.3390/biom11070923 34206421 PMC8301948

[B61] Zhang F. Xie J. Wang G. Zhang G. Yang H. (2018). Anti-osteoporosis activity of Sanguinarine in preosteoblast MC3T3-E1 cells and an ovariectomized rat model. J. Cell Physiol. 233 (6), 4626–4633. 10.1002/jcp.26187 28926099

[B62] Zhang W. L. Meng H. Z. Yang M. W. (2015). Regulation of DMT1 on bone microstructure in type 2 diabetes. Int. J. Med. Sci. 12 (5), 441–449. 10.7150/ijms.11986 26078704 PMC4466406

[B63] Zhang Y. Swanda R. V. Nie L. Liu X. Wang C. Lee H. (2021). mTORC1 couples cyst(e)ine availability with GPX4 protein synthesis and ferroptosis regulation. Nat. Commun. 12 (1), 1589. 10.1038/s41467-021-21841-w 33707434 PMC7952727

[B64] Zhao T. Yu Z. Zhou L. Wang X. Hui Y. Mao L. (2022). Regulating Nrf2-GPx4 axis by bicyclol can prevent ferroptosis in carbon tetrachloride-induced acute liver injury in mice. Cell Death Discov. 8 (1), 380. 10.1038/s41420-022-01173-4 36071041 PMC9452542

[B65] Zheng J. Woo S. L. Hu X. Botchlett R. Chen L. Huo Y. (2015). Metformin and metabolic diseases: a focus on hepatic aspects. Front. Med. 9 (2), 173–186. 10.1007/s11684-015-0384-0 25676019 PMC4567274

