# Supplementary material for: Metformin attenuates diabetic osteoporosis by suppressing ferroptosis via the AMPK/Nrf2 pathway
Source: Front Pharmacol. 2025 Mar 26;16:1527316. doi: 10.3389/fphar.2025.1527316 (PMC11979264; doi:10.3389/fphar.2025.1527316)
Supplement: Supplementary file 1 [file DataSheet1.docx]

Supplementary Material

**Fig. S1** Verification of the DOP rat model

**Fig. S2** Semi-quantitative analysis of GPX4 and SLC7A11 expression *in vivo*

**Fig. S3** Semi-quantitative WB analysis of different proteins expression in bone tissue

**Fig. S4** Semi-quantitative IHC analysis of ferroptosis-associated proteins and p-AMPK in bone tissue

**
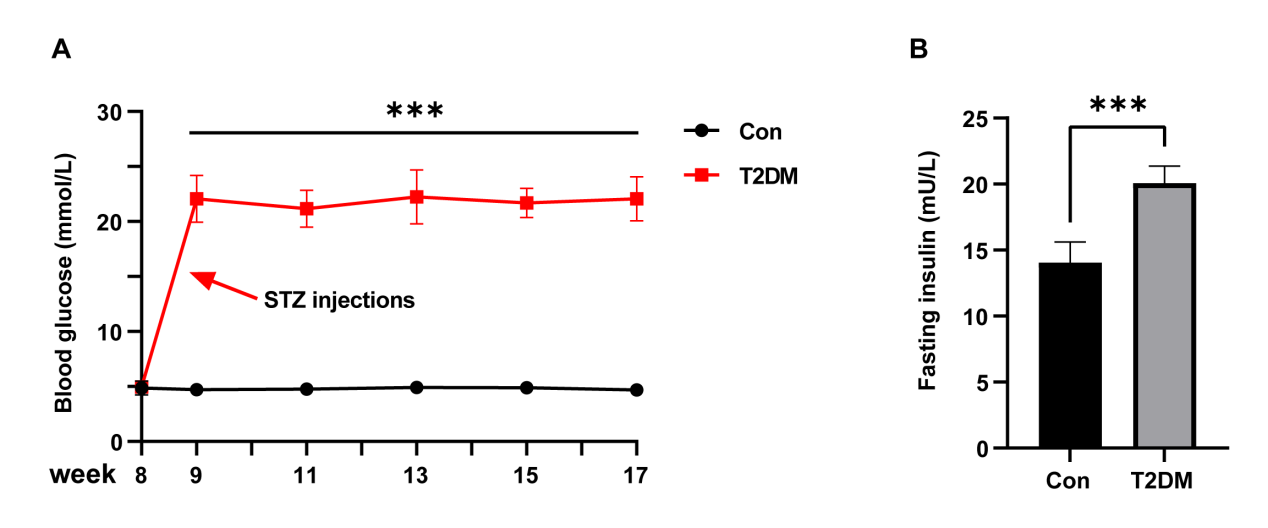
**

**Fig. S1** Verification of the DOP rat model. The DOP model was established using a high-fat diet combined with low-dose STZ injections. **(A)** Blood glucose levels were monitored every two weeks. **(B)** Fasting insulin levels were measured one week after STZ injection. ^*^*P* < 0.05, ^**^*P* < 0.01, ^***^*P* < 0.001. Each group contained six rats.

**
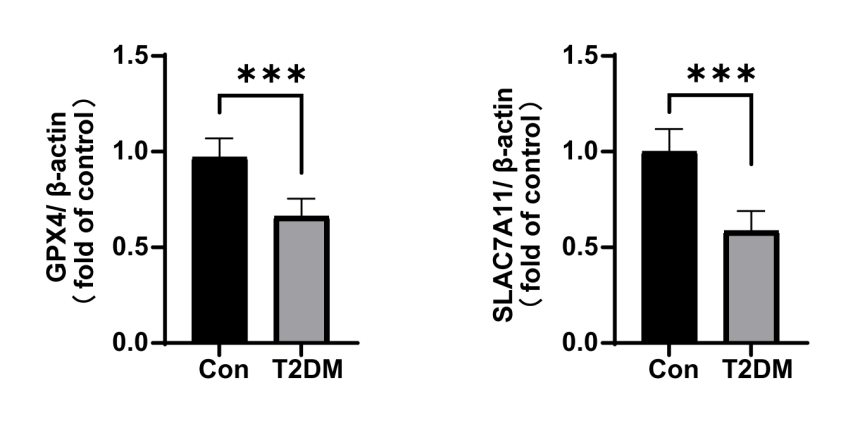
­­**

**Fig. S2** Semi-quantitative analysis of GPX4 and SLC7A11 expression *in vivo*. ^*^*P* < 0.05, ^**^*P* < 0.01, ^***^*P* < 0.001. Each group contained six rats.

^
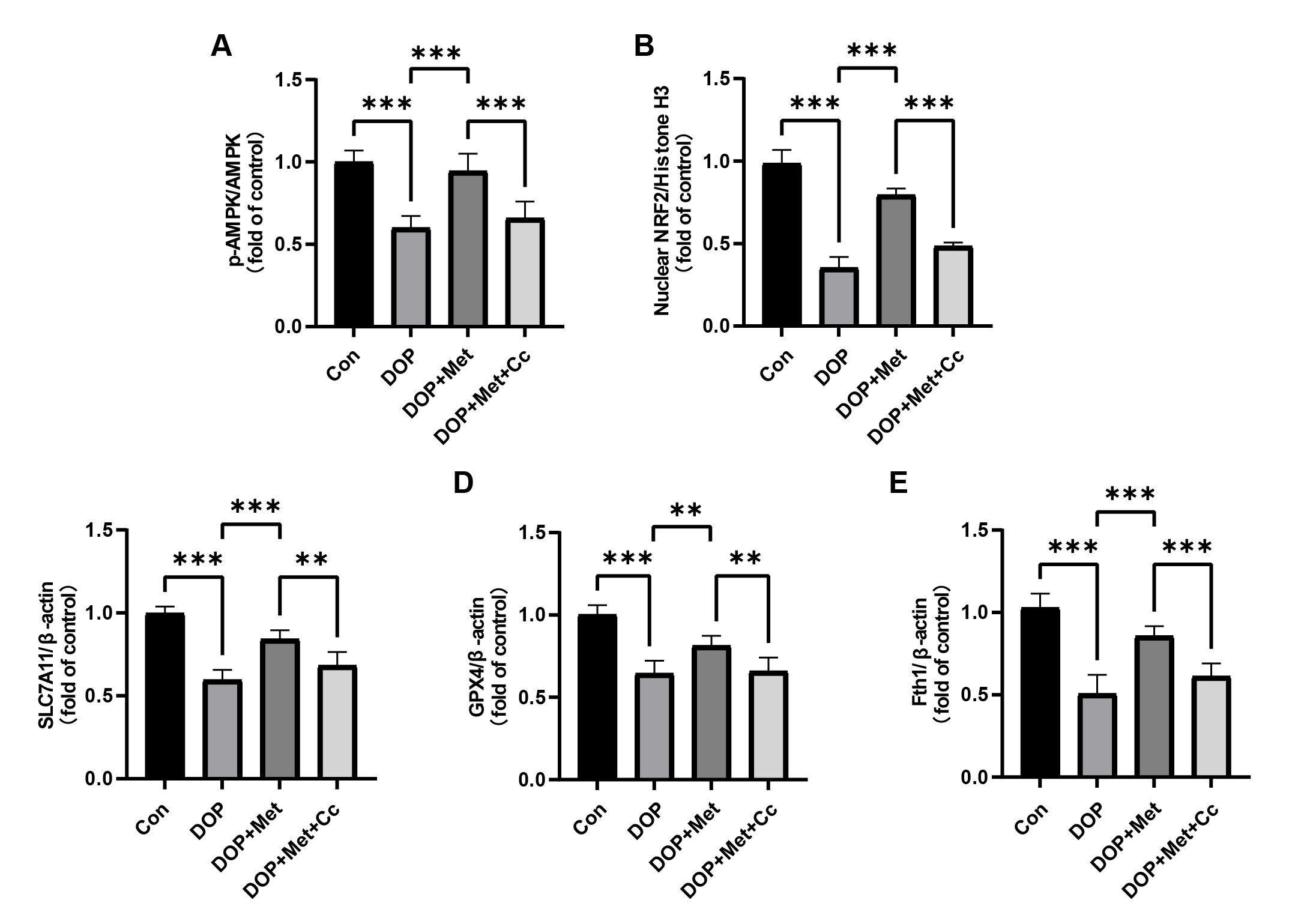
^

**Fig. S3** Semi-quantitative WB analysis of different proteins expression in bone tissue. ^*^*P* < 0.05, ^**^*P* < 0.01, ^***^*P* < 0.001. Each group contained six rats.

^
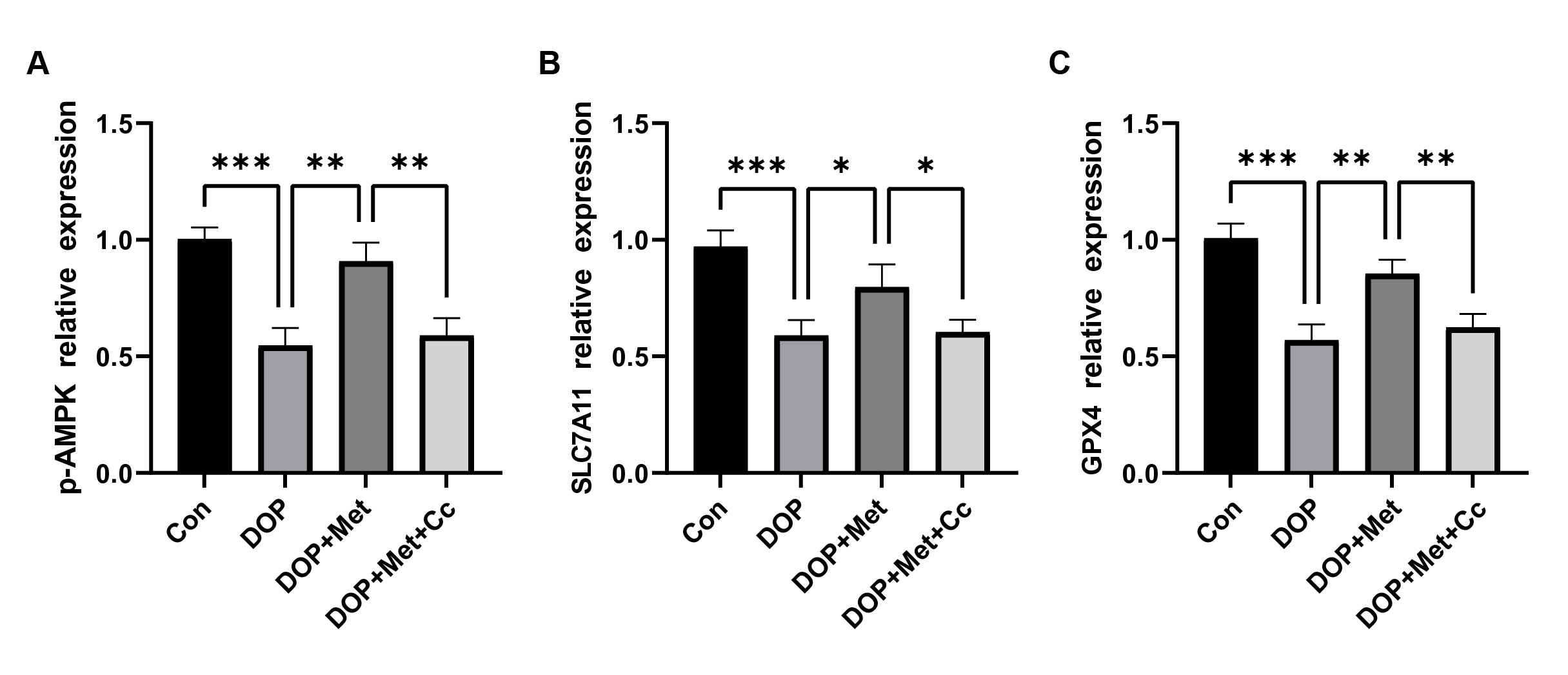
^

**Fig. S4** Semi-quantitative IHC analysis of ferroptosis-associated proteins and p-AMPK in bone tissue. ^*^*P* < 0.05, ^**^*P* < 0.01, ^***^*P* < 0.001. Each group contained six rats.
